# Supplementary material for: Depression diagnosis from patient interviews using multimodal machine learning
Source: Front Psychiatry. 2025 Nov 27;16:1694762. doi: 10.3389/fpsyt.2025.1694762 (PMC12696486; doi:10.3389/fpsyt.2025.1694762)
Supplement: Supplementary file 1 [file DataSheet1.pdf]

Supplementary Material

1 SUPPLEMENTARY TABLES AND FIGURES

1.1 Tables

Table S1: Overview of Covarep acoustic features.

Feature Name	Description
F0	Fundamental frequency (pitch) of the voice.
VUV	Voiced/unvoiced decision.
NAQ	Normalized Amplitude Quotient, a glottal flow measure.
QOQ	Quasi-Open Quotient, related to glottal opening.
H1H2	Difference in amplitude between the first and second harmonics.
PSP	Peak Slope Parameter, reflects harmonic richness.
MDQ	Maxima Dispersion Quotient, a measure of glottal asymmetry.
peakSlope	Slope of the harmonic peaks.
Rd	Glottal shape parameter.
Rd_conf	Confidence measure for the Rd parameter.
creak	Creaky voice probability.
MCEP_0-24	Mel cepstral coefficient 0-24 (spectral envelope).
HMPDM_0-24	Harmonic model phase distortion mean 0-24.
HMPDD_0-12	Harmonic model phase distortion deviation 0-12.

Table S2: Overview of formant features.

Feature Name	Description
F1	First formant – lowest vocal tract resonance frequency.
F2	Second formant – tongue position and vowel quality.
F3	Third formant – vocal tract shape.
F4	Fourth formant – high-frequency resonance.
F5	Fifth formant – additional vocal tract characteristics.

Table S3: Overview of text-based features.

Feature Name	Description
TTR	The type-token-ratio, the ratio of unique words (types) to the total number of words (tokens) in a text.
avg_sentence_length	The average number of words per sentence.
past_tense_ratio	The proportion of verbs in past tense relative to all verbs in the text.
pronoun_count	The total number of personal pronouns in the text.
mean_local_coherence	The average semantic similarity between adjacent sentences or clauses (often computed using embeddings).
filler_word_count	The number of non-content words used to fill pauses (e.g., ”um”, ”uh”, ”like”, ”you know”).
sentiment	A numeric or categorical measure of emotional tone (e.g., positive, negative, neutral), often derived using sentiment analysis tools.
